# Successful conservative treatment of placenta accreta with traditional Chinese medicine: A case report

**DOI:** 10.1097/MD.0000000000024820

**Published:** 2021-02-19

**Authors:** Huamin Huang, Jialin Wang, Keqin Li, Hongbo Ma

**Affiliations:** a First College of Clinical Medicine, Shandong University of Traditional Chinese Medicine, Jinan, Shandong; b The Affiliated Hospital of Shandong Academy of Chinese Medicine; c Shandong Provincial Hospital Affiliated to Shandong University, Jingwuweiqi Road, Jinan, Shandong, China.

**Keywords:** conservative treatment, placenta accreta, TCM

## Abstract

**Rationale:**

Currently, placenta accreta treatment mainly includes nonconservative surgical and conservative treatments such as Traditional Chinese medicine (TCM). This report describes the case of a 37-year-old woman who suffered incomplete placenta accreta after vaginal delivery and was cured by TCM. TCM treatment of placenta accreta has its own unique advantages, including low toxicity and few side effects, unaffected breastfeeding, and retention of the uterus, which can ensure the expulsion of residual placenta and be beneficial to patients’ physical and mental health.

**Patient concerns:**

Symptoms included a small amount of vaginal bleeding and occasional lesser abdominal pain. The patient showed lesser abdominal tenderness, a red tongue moss with petechial hemorrhage, and a hesitant pulse. The reproductive history was G3P2L2A1. In addition, the patient was afraid of having her uterus removed due to incomplete placental separation.

**Diagnoses:**

The case was diagnosed as placental accreta. Ultrasound is the preferred method of diagnosis, and biomarkers, such as beta hCG, assist in screening for placental accreta. Doppler ultrasonography showed that in the bottom of the right uterine cavity, there was an uneven echo group of 7.6 × 4.6 cm, which was not clearly demarcated from the posterior wall; the muscle layer became thinner, with a thinnest part of 0.19 cm, and abundant blood flow signals were observed (Fig. 1

**Figure 1:**
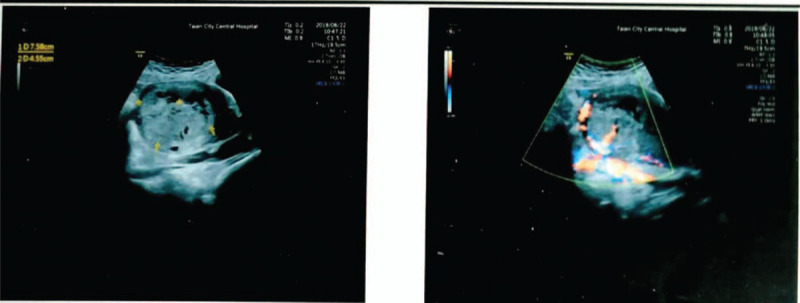
Ultrasound showing an uneven echo group of 7.6 × 4.6 cm in the bottom of the right uterine cavity, which was not clearly demarcated from the posterior wall. The muscle layer became thinner, with a thinnest part of about 0.19 cm. Abundant blood flow signals were observed.

). The beta hCG was 580.92 mIu/ml.

**Interventions:**

The patient initially underwent curettage therapy 9 days after delivery, but it failed due to excessive intraoperative bleeding. The patient then turned to TCM treatment. The doctor prescribed a multi-herbal formula.

**Outcomes:**

After 4 months, the residual placenta was expelled, and the patient's symptoms disappeared completely. No adverse and unexpected events occurred during treatment. During 3 months of follow-up, the patient had no abdominal pain, abnormal vaginal bleeding, or other complications.

**Lessons:**

This study shows that TCM is safe and effective for treating placenta accreta, and it is worth recommending TCM as a conservative treatment along with other treatments. In practice, however, we find that the earlier TCM treatment is applied, the better the effect; therefore, early intervention with TCM is particularly important.

## 1 Introduction

Placenta accreta is defined as the invasion of placental villi deep into the myometrium to varying degrees. Placenta accreta is often associated with factors such as endometrial injury, advanced maternal age, number of pregnancies, and increasing parity. It can result in massive hemorrhaging and the risk of complications, such as disseminated intravascular coagulation (DIC), multiorgan dysfunction, and/or failure and death.^[1]^ Currently, the treatment of placenta accreta mainly includes nonconservative surgical and conservative treatments, such as Traditional Chinese medicine (TCM).^[2]^ According to TCM, the pathogenesis of this disease involves impairment of the Chong and Ren Meridians, deficiency in the uterine collateral, placenta retention after childbirth, and uterine blood stasis. If not treated in time, placenta accreta may lead to secondary infections, perforation, or hemorrhaging of the pelvic cavity. Therefore, TCM is a promising therapy for placenta accreta worthy of being promoted and implemented worldwide.

## 2 Case report

**Figure 2:**
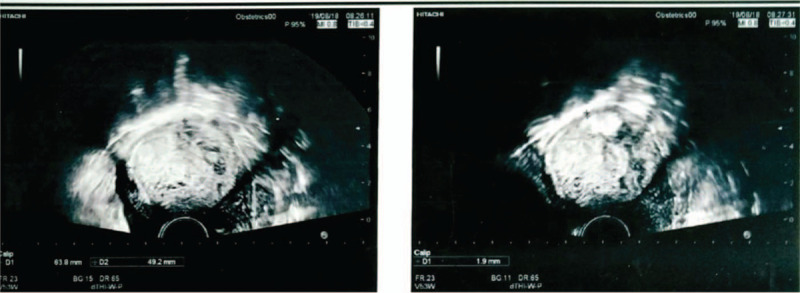
On the third day after curettage, ultrasound revealed an uneven echo group of 6.4 × 4.9 × 5.4 cm in the bottom of the right uterine cavity, which was not clearly demarcated from the posterior wall. The muscle layer became thinner, with a thinnest part of about 0.19 cm. Punctate blood flow signals were observed.

**Figure 3:**
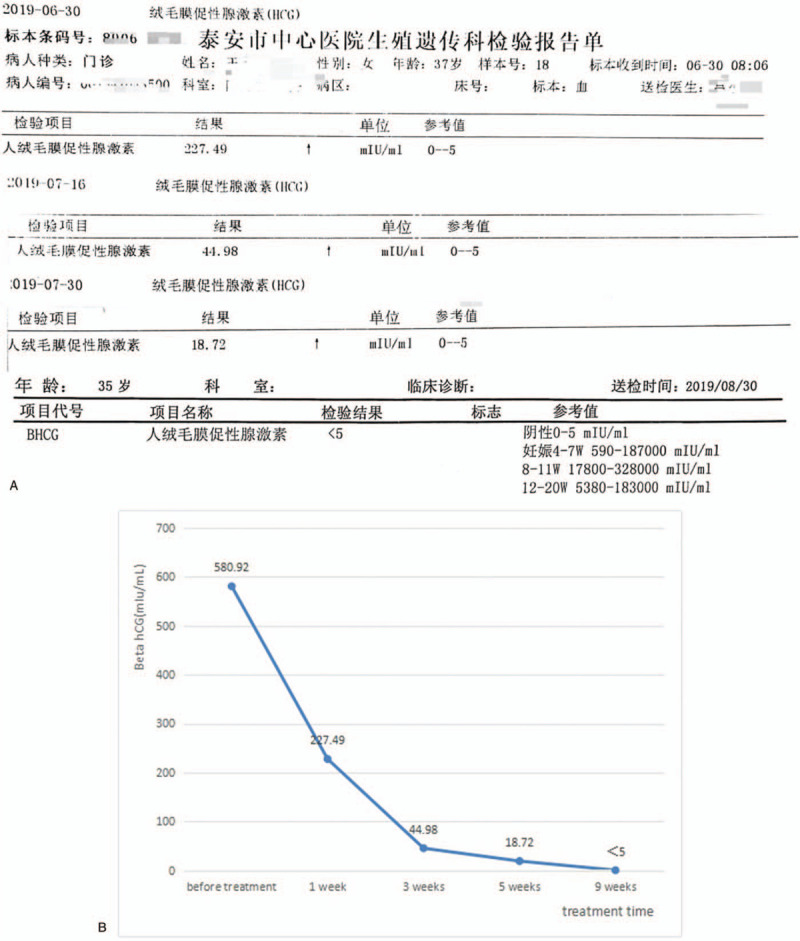
(A). Beta hCG records during treatment. (B). Beta hCG decreases with treatment duration.

**Figure 4:**
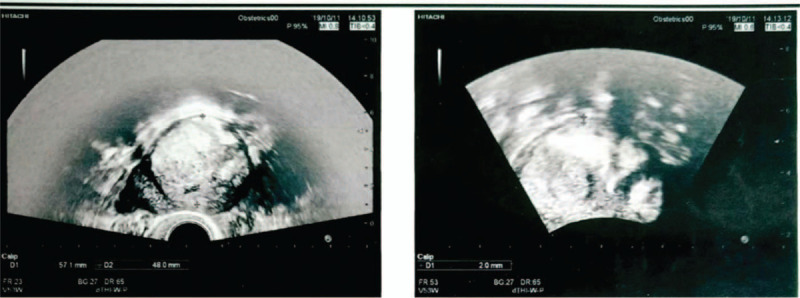
After more than 2 months of treatment, ultrasound indicated a 5.7 × 4.8 × 5.2 cm uneven echo group at the bottom of the uterine cavity, with a strong local echo and no obvious blood flow signal; the outer edge was about 0.2 cm from the thinnest part of the serous layer.

**Figure 5:**
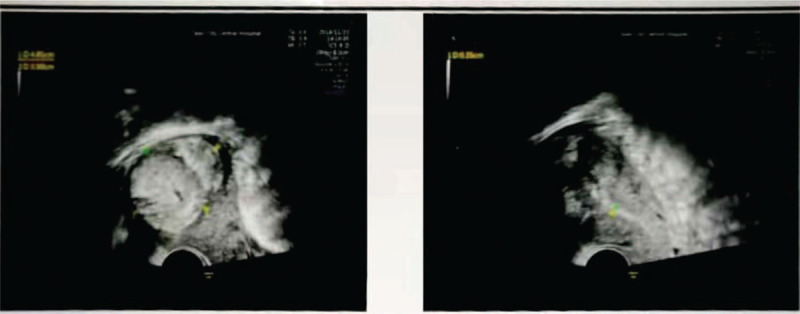
Three months later, ultrasound showed that the endometrial thickness of the middle and lower segment was about 0.35 cm, and that there was a 4.9 × 3.9 cm uneven echo group at the bottom of the uterine cavity, with a strong local echo and no obvious blood flow signal; the outer edge was about 0.24 cm from the thinnest part of the serous layer.

**Figure 6:**
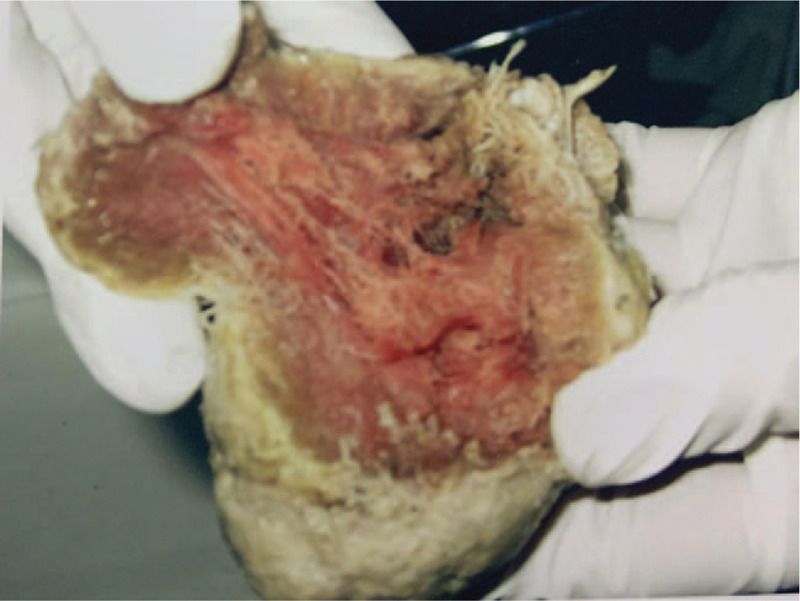
After taking the TCM continuously for 4 months, the placental tissue was expelled.

A 37-year-old pregnant woman with a history of 3 gravida, 1 parity, and 1 abortion, presented at 36 weeks and 2 days gestation with a vaginal delivery at Tai’an City Central Hospital on June 18, 2019. After delivery, she suffered from incomplete placental separation, accompanied by minor vaginal bleeding and occasional lesser abdominal pain. Ultrasound indicated an uneven echo group of 7.6 × 4.6 cm at the bottom of the right uterine cavity, which was not clearly demarcated from the posterior wall; the muscle layer became thinner, with a thinnest part of about 0.19 cm. Abundant blood flow signals were indicated (Fig. 1). Beta hCG was 2411.55 mIu/ml. The patient had been diagnosed with placenta accreta.^[3–4]^ The patient underwent curettage 9 days after delivery but had to be stopped due to excessive intraoperative bleeding. On the 3rd day after curettage, an ultrasound revealed an uneven echo group of 6.4 × 4.9 × 5.4 cm in the bottom of the right uterine cavity, which was not clearly demarcated from the posterior wall; the muscle layer became thinner, with the thinnest part about 0.19 cm, and punctate blood flow signals were indicated (Fig. 2). Beta hCG was 580.92 mIu/ml. The patient was lactating when she came to our hospital and expected conservative treatment. The symptoms were lesser abdominal pain and vaginal bleeding. The patient exhibited lesser abdominal tenderness, a red tongue moss with petechial hemorrhage, and a hesitant pulse. Therefore, a specific herbal formula was prescribed to eliminate the implanted placenta. The patient continuously took 1 dose per day, divided into 2 doses, of the medicine. The formula consisted of 15 g Sparganii Rhizoma, 15 g Curcuma Zedoary, 15 g of Cortex Moutan, 15 g Radix Paeoniae Rubra, 15 g Radix Salviae Miltiorrhizae-rhizae, 10 g Eupolyphaga seu Steleophaga, 12 g Radix Angelicae Sinensus, 12 g Semen Persicae, 12 g Flos Carthami, 15 g Pollen Typhae, 12 g Chuanxiong rhizome, 15 g Radix Cyathulae, 30 g Herba Taraxaci, 12 g Rhizoma Anemarrhenae, 15 g Radix Scutellariae, 12 g Radix Ophiopogonis, 12 g Radix Rehmanniae, 10 g Carapax Trionycis, 20 g Radix Trichosanthis, 30 g Herba Leonuri, 12 g Semen Vaccariae, and 6 g Medulla Tetrapanacis. After 1 week, vaginal bleeding stopped. Beta hCG was 227.49 mIu/ml. A month later, beta hCG was 18.72 mIu/ml. After more than 2 months, beta hCG was under 5 mIu/ml (Fig. 3). Ultrasound indicated a 5.7 × 4.8 × 5.2 cm uneven echo group at the bottom of the uterine cavity, with a strong local echo and no obvious blood flow signal; the outer edge was about 0.2 cm away from the thinnest part of the serous layer (Fig. 4). Three months later, ultrasound showed that the thickness of the endometrium in the middle and lower segments was about 0.35 cm, and there was a 4.9 × 3.9 cm uneven echo group at the bottom of the uterine cavity, with a strong local echo and no obvious blood flow signal; the outer edge was about 0.24 cm away from the thinnest part of the serous layer (Fig. 5). After taking the medicine continuously for 4 months, the patient had vaginal bleeding and paroxysmal abdominal pain, and the placental tissue was subsequently expelled (Fig. 6). No adverse and unexpected events occurred during the treatment. The doctor encouraged the patient to pay attention to their diet and maintain a positive attitude. After a 3-month follow-up, the patient had no lesser abdominal pain or abnormal vaginal bleeding.

## 3 Discussion

At present, there is no uniform standard for the treatment of placenta accreta. The protocol mainly includes nonconservative surgical treatments, such as hysterectomy, and conservative treatments. Cesarean hysterectomy is considered the gold-standard treatment for invasive accreta.^[5]^ It effectively guarantees the safety of the puerpera, although a loss of fertility may result. However, the risk of complications from hysterectomy has also been reported.^[6–7]^ Nowadays, most patients choose conservative treatments that preserve the uterus, which could preserve the fertility of patients and reduce psychological issues. Four different primary methods of conservative management have been described in the literature:

1. the extirpative technique (manual removal of the placenta);
2. the expectant approach (leaving the placenta in situ);
3. one-step conservative surgery (removal of the accretal area); and
4. the Triple-P procedure (suturing around the accretal area after resection).^[5]^

Methotrexate (MTX), a common adjunct to conservative treatment of placenta accreta, has been evaluated in some studies as having an additive effect in the conservative treatment of placenta accreta. However, there is not enough evidence about its efficacy and safety to recommend its routine use in all cases of invasive placenta.^[8]^ Due to an absence of other superior drugs, MTX is still widely used in clinical practice. In recent years, TCM has made some progress in the treatment of placenta accreta^[2]^ as a successful conservative treatment. This case report strongly supports the use of TCM to optimize the treatment of placenta accreta.

According to TCM, the pathogenesis of placenta accreta is due to the impairment of the Chong and Ren Meridians, deficiency in the uterine collateral, placenta retention after childbirth, and uterine blood stasis. Therefore, treatments involve removing blood stasis by promotion of blood circulation, clearing heat, and replenishing yin. The prescription is the E Leng Yuan Kun agent. Professer Keqin Li, a famous TCM expert from Shandong Province, created an empirical formula. The prescription was derived from modified amber powder, which was recorded in the Pu Ji Ben Shi Fang. It mainly consists of Stasis-Dispelling Blood-Quickening Medicinals and Heat-Clearing Toxin-Resolving Medicinals, which could promote the removal of residual placental tissue. Sparganii Rhizoma, Curcuma Zedoary, Cortex Moutan, Radix Paeoniae Rubra, Radix Salviae Miltiorrhizaerhizae, Eupolyphaga seu Steleophaga, Radix Angelicae Sinensus, Semen Persicae, Flos Carthami, Pollen Typhae, Chuanxiong rhizome, and Herba Leonuri are Stasis-Dispelling Blood-Quickening Medicinals; Herba Taraxaci, Rhizoma Anemarrhenae, and Radix Scutellariae are Heat-Clearing Toxin-Resolving Medicinals; and Radix Ophiopogonis and Radix Rehmanniae are Yin-Supplementing Medicinals. Studies of the molecular biology and pathology of placenta accreta suggest that inflammation and placental invasion may be closely related. Chronic basal inflammation combined with a failure of normal placental apoptosis appear to partially explain the underlying biology of invasive placentation with associated angiogenesis.^[9]^ Reports on placenta in situ have indicated that wide-spectrum antibiotics given after surgery are more effective.^[10]^ Stasis-Dispelling Blood-Quickening Medicinals can improve blood flow, promote the absorption of inflammatory exudate, and inhibit tissue dysplasia. Consequently, the absorption and expulsion of placental tissues are promoted.^[11]^ “Heat toxicity” can result in inflammatory reactions, which provides the theoretical basis for treating inflammation with Heat-Clearing Toxin-Resolving Medicinals.^[12]^ These medicinals have obvious advantages in reducing the risk of rebleeding and the use of antibiotics. Yin-Supplementing Medicinals can promote local blood circulation and are anti-inflammatory. Trichosanthin (TCS), an active component isolated from Radix Trichosanthis, can selectively damage placental trophoblasts. One study suggested that TCS could safely and effectively reduce levels of beta hCG, and that TCS could reach or exceed the success rate of MTX treatment.^[13]^ TCS could effectively reduce vaginal bleeding, accelerate the separation of the placenta from the uterine wall, and promote the expulsion of residual placental tissue.^[14]^ In addition, Semen Vaccariae and Medulla Tetrapanacis function to activate blood circulation to promote lactation. This herbal combination plays an important role in removing blood stasis by promoting blood circulation, clearing heat, and replenishing yin. In our practice, TCM has unique advantages for treating placenta accreta, including myometrium repair, placental villus tissue elimination, and infection prevention. Furthermore, there are fewer toxic side effects, and lactation is unaffected. Most importantly, the patient's fertility was retained, which was conducive to their physical and mental health. However, TCM has 1 drawback: it may not taste good. Therefore, we could try to change the formulation. During treatment, we monitored beta hCG, reexamined the ultrasound, and closely observed changes in the condition. After about 4 months of treatment, the residual placenta was removed, and beta hCG was under 5 mIu/ml. Therefore, we concluded that TCM is an effective and safe treatment for placenta accreta and worth recommending as a conservative treatment along with other medical interventions. In addition, we recommend early use of TCM therapy because the earlier it is used, the shorter the treatment time will be.

## Author contributions

Hongbo Ma designed the research; Huamin Huang and Jialin Wang performed the research; and Huamin Huang and Jialin Wang wrote the paper. All authors have read and approved the final version for publication.

**Data curation:** Huamin Huang, Jialin Wang.

**Investigation:** Huamin Huang, Jialin Wang.

**Methodology:** Keqin Li.

**Project administration:** Hongbo Ma.

**Writing – original draft:** Huamin Huang, Jialin Wang.

**Writing – review & editing:** Hongbo Ma.
